# Dual brain stimulation enhances interpersonal learning through spontaneous movement synchrony

**DOI:** 10.1093/scan/nsaa080

**Published:** 2020-06-15

**Authors:** Yafeng Pan, Giacomo Novembre, Bei Song, Yi Zhu, Yi Hu

**Affiliations:** School of Psychology and Cognitive Science, Institute of Brain and Education Innovation, East China Normal University, 200062 Shanghai, China; Neuropsychology and Functional Neuroimaging Research Unit (UR2NF), Université Libre de Bruxelles, B-1050 Bruxelles, Belgium; Department of Clinical Neuroscience, Karolinska Institutet, 17165 Stockholm, Sweden; Neuroscience and Behaviour Laboratory, Istituto Italiano di Tecnologia, 00161 Rome, Italy; Department of Neuroscience, Physiology and Parmacology, University College London, WC1E 6BT London, UK; School of Psychology and Cognitive Science, Institute of Brain and Education Innovation, East China Normal University, 200062 Shanghai, China; Department of Musicology, Harbin Conservatory of Music, 150070 Heilongjiang, China; School of Psychology and Cognitive Science, Institute of Brain and Education Innovation, East China Normal University, 200062 Shanghai, China; School of Psychology and Cognitive Science, Institute of Brain and Education Innovation, East China Normal University, 200062 Shanghai, China

**Keywords:** social interactive learning, inter-brain synchronization, spontaneous movement, music, dual brain stimulation

## Abstract

Social interactive learning denotes the ability to acquire new information from a conspecific—a prerequisite for cultural evolution and survival. As inspired by recent neurophysiological research, here we tested whether social interactive learning can be augmented by exogenously synchronizing oscillatory brain activity across an instructor and a learner engaged in a naturalistic song-learning task. We used a dual brain stimulation protocol entailing the trans-cranial delivery of synchronized electric currents in two individuals simultaneously. When we stimulated inferior frontal brain regions, with 6 Hz alternating currents being in-phase between the instructor and the learner, the dyad exhibited spontaneous and synchronized body movement. Remarkably, this stimulation also led to enhanced learning performance. These effects were both phase- and frequency-specific: 6 Hz anti-phase stimulation or 10 Hz in-phase stimulation, did not yield comparable results. Furthermore, a mediation analysis disclosed that interpersonal movement synchrony acted as a partial mediator of the effect of dual brain stimulation on learning performance, i.e. possibly facilitating the effect of dual brain stimulation on learning. Our results provide a causal demonstration that inter-brain synchronization is a sufficient condition to improve real-time information transfer between pairs of individuals.

## Introduction

Learning through interactions with others is one of the most extraordinary skills of humans among other social species (Chen *et al.,* 2016; Verga and Kotz, 2019). Learning new information from a conspecific is often indispensable for survival. Yet, the scientific study of social interactive learning, and its underlying neurophysiological processes, has begun only recently (Pan *et al.,* 2018, 2020; Zheng *et al.,* 2018; Liu *et al.,* 2019).

A fundamental prerequisite of social interactive learning is the presence of (at least) two individuals: one teaching something to another. Accordingly, the most recent brain research in this area is moving towards paradigms entailing the simultaneous recording of two individuals’ neural activity, and the analysis of their inter-dependency (Redcay and Schilbach, 2019). This is generally referred to as ‘second-person neuroscience’ (Schilbach *et al.,* 2013; Redcay and Schilbach, 2019) or more specifically ‘hyperscanning’ (Montague *et al.,* 2002; Dumas *et al.,* 2011; Hasson *et al.,* 2012; Babiloni and Astolfi, 2014; Hasson and Frith, 2016).

In a recent hyperscanning study, we examined brain activity from dyads composed of instructors and learners engaged in the acquisition of a (music) song (Pan *et al.,* 2018). We observed that neural activity recoded over the inferior frontal cortices (IFC) of the instructor and the learner become synchronized, particularly when the learner was observing the instructor’s behavior. Remarkably, inter-brain synchronization (IBS) predicted learning performance, in particular the learner’s accuracy in pitch learning performance (i.e. intonation).

Our observations join others in suggesting that interpersonal synchronous brain activity might be a correlate of social behavior (Liu *et al.,* 2017; Nastase *et al.,* 2019; for a review, see Hasson *et al.,* 2012), including social interactive learning (Pan *et al.,* 2018, 2020; Zheng *et al.,* 2018; Bevilacqua *et al.,* 2019; Liu *et al.,* 2019). Yet, the functional significance of this phenomenon remains elusive. One could claim that synchronous brain activities occur as a consequence of social interactive learning. Alternatively, a stronger claim could suggest that IBS causally enhances learning. If this was the case, then it should be predicted that exogenously enhancing IBS would enhance learning performance.

To test the above hypothesis, we adopted a ‘dual brain stimulation’ protocol (Novembre *et al.,* 2017). This consists of simultaneous electric currents delivered trans-cranially in two individuals simultaneously. By manipulating the coupling between the signals delivered across two brains, the experimenters can control IBS and monitor its causal effects upon social behavior (Novembre *et al.,* 2017).

**Fig. 1:**
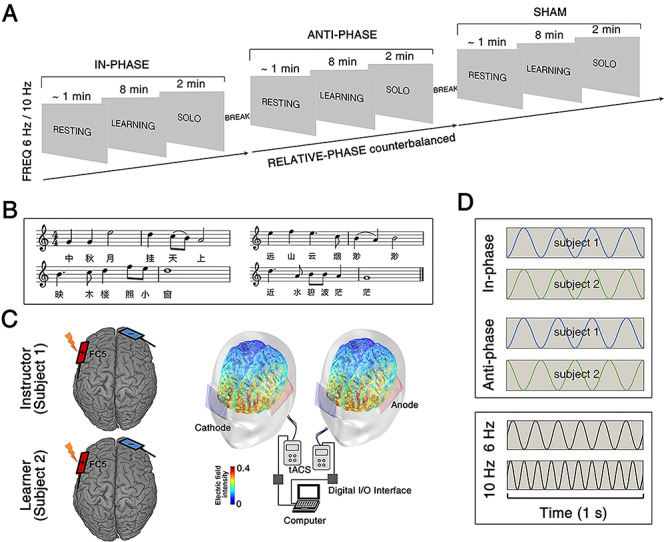
Experimental task and design. (A) The social interactive learning task consisted of three sessions: resting, learning and solo. (B) Representative musical score used in the social interactive learning task. (C) Schematic illustration of the brain stimulation montage. Left: dual brain stimulation was administered through simultaneous tACS to the instructor and the learner. The electrodes were placed over FC5 (anode) and FP2 (cathode), according to the international 10/10 system, in order to best target the left inferior frontal cortices. Right: electric field simulation shows that this montage entrains neural activity in the brain region of interest. (D) The dual brain stimulation protocol entails manipulations of relative-phase (between instructor and learner) in different frequencies.

Following up on our previous study (Pan *et al.,* 2018), we targeted the IFCs of dyads composed of an instructor and a learner—engaged in the acquisition of a song—using pairs of transcranial alternating current stimulators (tACS). We delivered alternating currents oscillating in the theta frequency range (6 Hz) because oscillations in this band are commonly observed over the frontal cortex, specifically in the context of tasks requiring auditory (including music) processing and learning (including vocal learning) (Assaneo *et al*., 2019; Behroozmand *et al.,* 2015; Crivelli-Decker *et al.,* 2018; Hsiao *et al.,* 2009; Lindenberger *et al.,* 2009; Sänger *et al.,* 2012; Wischnewski *et al.,* 2016)—all prerequisites to our song-learning task. Crucially, we manipulated the relative phase of the learner’s and the instructor’s currents (Figure 1, lower panels), being these either perfectly in-phase (0° relative phase) or anti-phase (180° relative phase). For control purposes, we also included a control stimulation frequency of 10 Hz, as representative of the alpha band (Klimesch, 2012), and a sham stimulation condition.

We collected two distinct measures of behavior. On the one hand, we asked a group of expert raters to evaluate how well had the learners acquired the musical material. In line with our previous work, we expected 6 Hz in-phase dual brain stimulation to enhance intonation learning performance (Novembre *et al.,* 2017; Pan *et al.,* 2018). On the other hand, we used HD video recordings of the learning task to extract indices of spontaneous movement of the two participants. This second measure was meant to be exploratory. It was inspired by a growing body of research indicating that interpersonal synchronous movement can augment pro-social behaviors (Hove and Risen, 2009; Wiltermuth and Heath, 2009; Keller *et al.,* 2014; Endedijk *et al.,* 2015; Reddish *et al.,* 2016; Hu *et al.,* 2017), hypothetically even social learning.

## Methods

### Participants

We recruited 28 healthy, right-handed volunteers: 24 of which acted as learners (mean age ± s.d.: 20.96 ± 2.31, age range: 17–25), while the remaining four acted as music instructors (mean age ± s.d.: 19.25 ± 0.50, age range: 19–20). Participants were recruited via advertisements and flyers spread within the East China Normal University. We tested only female–female participant dyads in order to mitigate inter-individual and inter-dyad variability (Cheng *et al.,* 2015; Baker *et al.,* 2016), in accordance with recent work (Pan *et al.,* 2018, 2020; Tang *et al.,* 2019). The four instructors were required to have received at least 10 years of formal musical training, and they were all members of a local choir. The 24 learners were required to have (i) less than 3 years of formal musical training and (ii) no musical training at all within the past 5 years. Each of the four instructors was paired with six learners, in a one-by-one fashion, resulting in a total of 24 instructor-learner dyads. The sample size was determined in order to be consistent with our previous study (Pan *et al.,* 2018), which motivated the current one. Specifically, both studies included 24 dyads, a number that is generally in line with instructor-learner hyperscanning studies (Takeuchi *et al.,* 2017; Bevilacqua *et al.,* 2019; Pan *et al.,* 2020). Notably, in the current study we recruited a higher number of instructors (cf. Pan *et al.,* 2018) in order to mitigate instructor-specific effects. None of the participants had a history of neurological or psychiatric illness. All participants were naïve with respect to the purpose of the study. Each learner and each instructor was reimbursed with approximately U$8.5 and U$50 for their participation, respectively, (in local currency). Each participant provided informed consent prior to the experiment. The study was approved by the University Committee of Human Research Protection (HR 125–2018) from East China Normal University.

### Experimental task

In a social interactive learning task, the instructor taught three songs to each learner individually while seating face-to-face (0.8 meters apart). The instructor and the learner’s chairs were slightly oriented towards the camera to improve whole-body visibility (resulting in approximately a 90° angle in between the two chairs’ orientations). Each song was taught within a dedicated block, which comprised three sessions: resting, learning and solo (Figure 1A). During the resting session (~1 min), the instructor and the learner were asked to relax and to avoid unnecessary movement. During the following learning session (8 min), the instructor taught the song to the learner in a turn-taking manner; i.e. the learner attended and then imitated every single phrase of the song (i.e. one-by-one) performed by the instructor (Pan *et al.,* 2018). Note that the learning task was meant to unfold in a naturalistic manner, and therefore both the instructor and the learner were free to use vocal and non-vocal communication (including facial expressions or gestures) to facilitate the acquisition of the song. Finally, during the solo session (2 min), learners were instructed to sing the whole song as best as they could. This allowed us to record the final performance and later assess how well the song had been acquired.

### Musical material

We selected three Chinese songs conveying a similar musical structure (e.g. quadruple rhythm, eight bars and slow tempo) and emotion (i.e. missing home): (i) ‘The Moon Reflection’ (Lyrics: B. Peng, Music: Z. Liu and S. Yan), (ii) ‘Nostalgia’ (Lyrics: T. Dai, Music: Z. Xia) and (iii) ‘A Tune of Homesickness’ (Lyrics: C. Qu, Music: Q. Zheng) (Figure 1B displays a segment from ‘Nostalgia’). These songs were selected because they were meant to be unfamiliar to the learners (as confirmed by learners’ report) and because they were used in our previous work motivating the current study (Pan *et al.,* 2018).

### Experimental design

During (and only during) the learning session, we delivered tACS to both the learner and the instructor using a dual brain stimulation protocol (Figure 1C, see the next section for technical details). Our experimental design entailed two manipulations. First, we manipulated the FREQUENCY of the induced current, being 6 Hz for half of our participants and 10 Hz for the remaining half. Note that age, prior musical training (in years), pitch discrimination and music memory abilities (as assessed by a 6-min online test, http://jakemandell.com/tonedeaf/, Mandell *et al.,* 2007; **Table S1**) were comparable across these two groups (*t*s < 0.79, *P*s > 0.44). Second, we manipulated the RELATIVE-PHASE of the signals delivered across the instructors and the learner. These could be either in-phase (i.e. 0° relative phase) or anti-phase (i.e. 180° relative phase) (Figure 1D). A third sham stimulation condition was also included for control purposes, leading to a full 2 × 3 factorial design. The order of the RELATIVE-PHASE conditions, as well as the order of the selected songs, was fully counterbalanced and manipulated orthogonally (see Supplementary Table S2 for more details).

### Dual brain stimulation

A dual brain stimulation protocol was used to deliver simultaneous signals to the brains of the instructor and the learner during the learning session. To achieve this, we used two battery-driven tACS stimulators (Model: 2001; Soterix Medical Inc., New York, USA). The signals were delivered trans-cranially through two electrodes covered with rubber (5 × 5 cm; Soterix Medical Inc., New York, USA) and soaked in a saline solution (5 × 7 cm; Soterix Medical Inc., New York, USA). All stimulation electrodes were secured to the scalp with rubber head straps. For both participants, the anode electrode was placed over the left IFC (equivalent to electrode position FC5 according to the international 10/10 system), while the cathode electrode was placed over the contralateral frontopolar cortex (Figure 1C) (Holland *et al.,* 2011, 2016). An electric field simulation obtained using the COMETS Toolbox (version 2.0) (Lee *et al.,* 2017) confirmed that this montage is appropriate to entrain neural activity in the left IFC **(**Figure 1C**).**

The stimulation entailed a sinusoidal wave having a peak-to-peak amplitude of 1 mA, which ended the ramping up phase when the learning session began and began the ramping down phase when the session ended. The frequencies and relative phases adopted are described in the previous section. For sham stimulation conditions, both subjects received a 30 s fade-in followed by a 30 s fade-out of stimulation.

The two stimulators were controlled through a National Instruments Data Acquisition Toolbox Support Package (NI-DAQmx), which was controlled using MATLAB (MathWorks Inc., Natick, MA) via two USB/Parallel 24-Bit Digital I/O Interfaces (Model: SD-MSTCPUA; Cortech Solutions Inc., North Carolina, USA). The latter was connected to a computer running the Data Acquisition Toolbox as well as to each stimulator. An external trigger was sent simultaneously from the computer to the Digital I/O Interfaces so that the two stimulators could begin the stimulation at the same time (Figure 1C). An anonymous reviewer questioned whether the above setting would be sufficient to induce synchronous stimulations, and whether any gradual phase shift was observed throughout stimulation. These issues were ruled out in a control experiment that we report in the **Supplementary Text**.

Prior to the experiment, all participants were exposed to tACS for approximately 1 min to ensure they were comfortable with the stimulation intensity. Both the instructor and the learner were naïve with respect to the RELATIVE-PHASE and FREQUENCY conditions applied (see **Supplementary Text** for details). After each experimental block, participants were asked to report potential side effects of the tACS. They filled in a questionnaire including the following items: pain, burning, heat, itchiness, pitching, metallic taste, fatigue, skin flush, the effect on performance or any other side effects perceived. Reported side effects were comparable across conditions (*F*s < 1.14, *P*s > 0.12).

The instructors, who were meant to teach to six different learners and therefore undergo the stimulation procedure on six different occasions, were allowed to a maximum of two teaching sessions per week (with at least three days in between).

### Video and audio recordings

The whole procedure was video recorded using a fixed digital camera (HDR-CX610E, Sony, Tokyo, Japan). Recording files were stored using an MTS format. The room illumination was controlled in order to be stable and support optimal shutter speed and aperture. The distance between the camera and dyads was about 2 m so that both participants’ full bodies and faces could be captured. Additionally, a digital voice recorder (ICD-PX470, Sony, Tokyo, Japan) was used to record the vocalizations. The voice recorder was placed nearby the participants (~30 cm). The audio files were stored in WAV format. The high-quality video and audio recordings were subsequently used to quantify movement dynamics and evaluate the song learning performance.

### Data analysis

Two main analyses were conducted. First, the video data collected during the learning session were analyzed in order to quantify whether the learner and the instructor movements synchronized. Second, we analyzed both the video and the audio recordings associated with the solo session. These recordings were presented to a group of expert raters (naïve with regard to the purpose of the study), who rated how well the materials had been acquired. All of these analyses were firstly conducted within experimental conditions, and the results were later compared statistically (see next section).

#### Video analysis of the learning session

Preprocessing of the video data was conducted using the Format Factory (version 4.1.0, PCFreetime Inc., Shanghai, China). The MTS files were first converted into the MP4 format (FPS 25; dimension 1920 × 1080). The converted data (25 frames/s) were segmented according to the trial structure, i.e. separate segments, either associated with the learning session or with the solo session, were extracted. The analysis of the segments associated with the learning session consisted of the following four steps.

**Fig. 2:**
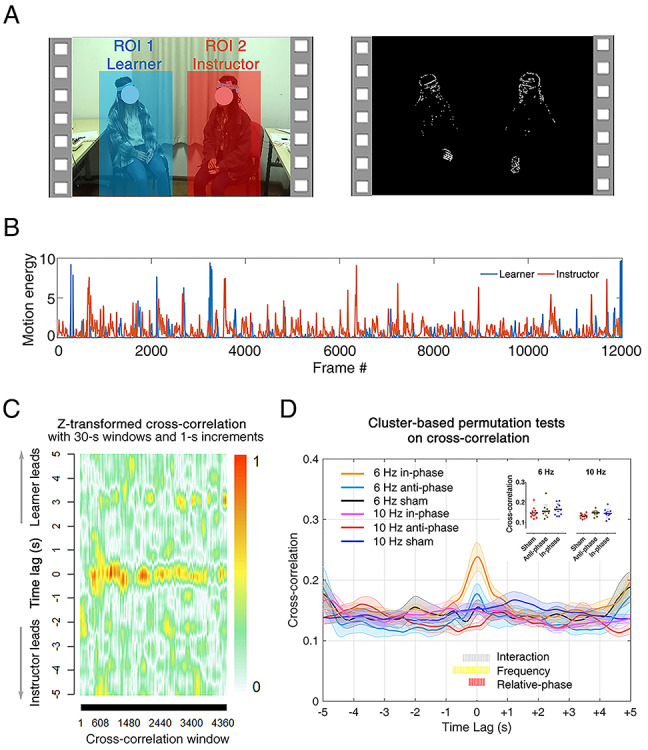
Motion energy extraction and cross-correlation analysis. (A) Regions of interest (ROI) utilized for the video-based MEA. ROI1 covers the face and body of the learner (in blue); ROI2 covers the face and body of the instructor (in red). (B) Representative motion energy time series. (C) Cross-correlation coefficients obtained using a moving window approach (window size = 30 s; moving in steps of 1 s) in a representative dyad from the 6 Hz group. (D) Cross-correlation coefficients averaged across time, and participants, within each condition. The shaded area denotes the standard error at each time lag. We used a cluster-based permutation test to control for multiple comparisons. Periods of time associated with significant clusters are marked on the bottom. Single dyads’ coefficients (averaged within the ‘interaction’ cluster) are plotted on the top-right side of the panel.

Step 1: Instructor and learner movement extraction. A motion energy analysis (MEA) algorithm was used to compute a continuous measure of movement associated with either the instructor or the learner (Ramseyer and Tschacher, 2011). This algorithm employs a frame-differencing approach; i.e. it quantifies the amount of change from one frame to the next, i.e. motion energy (Kupper *et al.,* 2015). The algorithm was applied to two separate regions of interest, each covering the full body of the instructor or the learner (Figure 2A).

Step 2: Preprocessing of time series. The motion energy signals resulting from the previous step were smoothed using a moving average window (span = 0.5 s). Next, outlying data points within the time series (i.e. values exceeding mean + 10 × STD of the time series, Kupper *et al*., 2015) were removed (1.23 ± 0.21% of the whole data).

Step 3: Cross-correlation analysis. The preprocessed time series were subsequently submitted to a cross-correlation analysis, which was meant to quantify the dynamic synchrony between the instructor and the learner. Before entering the data into this analysis, we controlled whether the mean and standard deviation of the time series (indexing the amount of movement and movement variability) were comparable across conditions (all *P* values >0.18). Next, motion energies associated to the instructor and the learner were cross-correlated, separately for each condition, using a moving window (span = 30 s, maximum lag = 5 s, step = 0.04 s, leading to 125 steps) (Tschacher *et al*., 2014). Note that the moving window approach is appropriate considering the non-stationary nature of movement behaviors.

Step 4: Interpersonal movement synchrony. Cross-correlation coefficients comprised 251 time lags (125 cross-correlations for positive lags, 125 for negative lags and 1 for the zero lag). These were Fisher’s z transformed to obtain a bivariate normal distribution. In line with previous studies (Tschacher *et al*., 2014), the coefficients were then turned into absolute values and averaged across the moving windows. Thus, the cross-correlation coefficients ranged from 0 to +1.

Data preprocessing and analyses were conducted in R (statistical environment, version 3.6.3; R Core Team, 2020) using the rMEA package and custom-made codes (Kleinbub and Ramseyer, 2018). In order to control for spurious synchrony, we additionally ran a pseudosynchrony analysis (i.e. comparing real associations found in genuine dyads with chance associations produced by shuffling the data; Kleinbub and Ramseyer, 2018; Ramseyer and Tschacher, 2011). The results of this control analysis confirmed that interpersonal movement synchrony did not occur by chance (for more details, see **Supplementary Text**).

#### Expert raters judging the solo session

The video and audio recordings associated with the solo session (2 min) were presented to a group of six postgraduate students majoring in musicology [all blind to the experiment’s purposes, all having at least 8 years (mean ± s.d. = 10.50 ± 3.39 years) of music training experience (i.e. implying music lessons and singing)]. These music students, as expert raters, were asked to evaluate how well the music pieces had been acquired by providing subjective ratings on the 7-point Likert scales. The ratings consisted of the following six aspects (adapted from Pan *et al.,* 2018; see **Table S3** for details): (i) *intonation*: pitch accuracy; (ii) *melody*: ability to accurately express the linear succession of musical tones; (iii) *rhythm*: effective expression of the timing of musical sounds and silences that occur over time; (iv) *lyric*: accuracy in singing the lyrics; (v) *emotion*: ability to effectively express the emotion of the song. Signs of high ability include emotional facial and vocal expression; (vi) *overall performance*: the overall ability to perform the music song. The ratings were on a 7-point Likert scale, ranging from 1 (‘very low’) to 7 (‘very high’). We averaged the ratings provided by different raters and confirmed that the final score had very high inter-rater reliability (intra-class correlation on six aspects = 0.704–0.960). Also note that the sum of the first five aspects (i.e. intonation + melody + rhythm + lyrics + emotion) was perfectly correlated with the last one (i.e. overall performance), *r* = 0.97, *P* < 0.001.

### Statistical tests

The data were analyzed using multilevel (mixed effects) modeling. A conventional approach using mixed-design analysis of variance was also attempted and provided analogous results. However, the modeling approach was deemed as more appropriate for the current dataset given the inter-dependencies of some data points (i.e. the fact that each instructor formed six dyads with six distinct learners).

The model was constructed using the *lme4* package in R (Bates *et al.,* 2015). The *lmerTest* was used to perform significance tests on the parameters yielded by the model (Kuznetsova *et al*., 2017). Data were modeled by RELATIVE PHASE (in-phase, anti-phase or sham), FREQUENCY (6 *vs* 10 Hz) and the interaction of these fixed effects. Random effects were estimated for LEARNER and INSTRUCTOR (with the former being nested within the latter). Next, we performed significance tests using Satterthwaite *P* values (Luke, 2017). For significant main effects or interaction, follow-up contrasts were further performed using the *emmeans* package in R (Lenth, 2018), with a Tukey adjustment to control for multiple comparisons.

When analyzing the cross-correlation coefficients, we conducted multilevel modeling for each of the 251 time lags. The results from this series of multilevel modeling (i.e. both the main effects and the interaction) required correction for multiple comparisons. To do so, we used a cluster-based permutation test (Maris and Oostenveld, 2007, see also Novembre *et al*., 2019 for a similar application of the test). The cluster-based permutation test is inspired by research in neurophysiology, where scholars normally apply the same test over signals composed of multiple consecutive time points (in our case, across ±5 s time lags; cf. Maris and Oostenveld, 2007), therefore requiring a correction for multiple comparisons. Specifically, adjacent time lags associated with a significant alpha level (*P* < 0.05) were grouped into a cluster. Then, a cluster-level statistic was calculated by taking the sum of the *F*-values within the observed clusters. The largest cluster was retained. To evaluate the significance of such largest cluster, we further created permuted data by shuffling dyads’ averages, i.e. randomly assigning dyads’ data to (i) either 6 or 10 Hz groups and, within dyads, to (ii) RELATIVE-PHASE conditions. The significance level was assessed by comparing the cluster statistics from the original data with 1000 renditions of permuted data using the Monte Carlo method (thresholded at *P* < 0.05).

Expert ratings were analyzed using the same multilevel modeling, one for each of the six aspects being evaluated. Significant (*P* < 0.05) main effects or interactions were followed up by pairwise comparisons. Tukey adjustment was used to account for *post hoc* multiple comparisons within each aspect of expert ratings.

Finally, we conducted correlation and mediation analyses to explore potential relationships between distinct dependent variables: interpersonal movement synchrony and expert ratings. In particular, following up on the results from the previous analyses, we computed ‘Δ interpersonal movement synchrony’, indexing the relative increase of interpersonal synchrony in the in-phase *vs* sham stimulation condition, as well as ‘Δ intonation learning performance’, indexing the relative enhancement of intonation performance following in-phase *vs* sham stimulation condition. Next, we conducted a Pearson correlation between these measures, as well as a mediation analysis, using INDIRECT macro (Preacher and Hayes, 2008) implemented in SPSS (version 18.0, SPSS Inc., Chicago, IL, USA). We constructed the mediation model based on the following regression equations:

(1) \begin{equation*} Y= cX+{e}_y, \end{equation*}

(2) \begin{equation*} M= aX+{e}_m, \end{equation*}

(3) \begin{equation*} Y=c{\prime}X+ bM+e{\prime}_y, \end{equation*}

where *X* is the independent variable (frequency of the dual brain stimulation, dummy coded, with 0 for 10 Hz and 1 for 6 Hz), *M* is the mediator (Δ interpersonal movement synchrony), *Y* is the dependent variable (Δ intonation learning performance) and *e* denotes residuals. Path *a* is the coefficient relating *X* to *M*. Path *b* is the coefficient relating *M* to *Y* adjusted for *X*. Paths *c’ and c* describe the relationship of *Y* and *X* with and without *M*, respectively, (see Figure 4B; see also Mac Kinnon *et al.,* 2007 for a review). A control analysis confirmed that the instructor-specific effect did not confound the mediation results (see **Supplementary Text**). Note that because the independent variable of the model is between-dyad, the results reflect whether the group difference in interpersonal movement synchrony could mediate the group difference in intonation learning. The bootstrapping method embedded in INDIRECT was used to determine whether the mediation effect was different from zero with 95% confidence. The number of bootstrap samples was set to 5000. Confidence intervals for indirect effect were bias corrected (Preacher and Hayes, 2008). Data are available at https://osf.io/bqsd5/.

## Results

The social learning task comprised two main sessions: a learning session and a solo session. During the learning session, the instructor taught the song to the learner while their brains were simultaneously stimulated. Next, during the solo session, learners were instructed to sing the newly acquired song as best as they could, while the performance was recorded in order to be evaluated by expert raters afterwards (Figure 1). For consistency with the temporal order of the two sessions, we firstly present the results of the interpersonal movement synchrony analysis (associated with the learning session) and later we report the experts’ ratings of the solo performances. Finally, we report correlation and mediation analyses addressing the relationship between interpersonal movement synchrony and learning performance.

### Dual brain stimulation enhanced interpersonal movement synchrony

The results from the cross-correlation analysis are displayed on Figure 2 (**C** and **D**). The cluster-based permutation test on these data yielded evidence in favor of a main effect of stimulation FREQUENCY (lags from −0.76 to +0.40 s, cluster-corrected *P* < 0.01), a main effect of RELATIVE-PHASE (lags from −0.24 to +0.24 s, cluster-corrected *P* < 0.01) and an interaction between these two (lags from −0.44 to +0.40 s, cluster-corrected *P* < 0.05). These results indicated that interpersonal movement synchrony was generally higher when the two brains were stimulated (*i*) at 6 Hz (mean ± s.d. of the cross-correlation coefficients, 0.18 ± 0.04), as opposed to 10 Hz (0.14 ± 0.02), *t*_(19)_ = 3.17, *P* = 0.005, and specifically (*ii*) in-phase (0.19 ± 0.07), as opposed to sham (0.13 ± 0.03), *t*_(44)_ = 4.46, *P* = 0.0002. Crucially, the significant interaction indicated that interpersonal movement synchrony (averaged within the significant ‘interaction’ cluster spanning between lag −0.44 to lag +0.40 s; Figure 2D) was specially stronger in the 6 Hz in-phase condition (0.22 ± 0.06), as opposed to the 6 Hz sham condition (0.15 ± 0.04), *t*_(44)_ = 4.81, *P* = 0.0001, and to the 10 Hz in-phase condition (0.15 ± 0.05), *t*_(54.6)_ = 4.06, *P* = 0.0002. In contrast, movement synchrony in the 6 Hz anti-phase condition (0.17 ± 0.04) neither significantly exceed that in the 6 Hz sham condition (0.14 ± 0.02), *t*_(44)_ = 2.07, *P* = 0.11, nor exceed that in the 10 Hz anti-phase condition (0.15 ± 0.03), *t*_(54.6)_ = 1.41, *P* = 0.16.

### Dual brain stimulation improved intonation learning performance

**Fig. 3:**
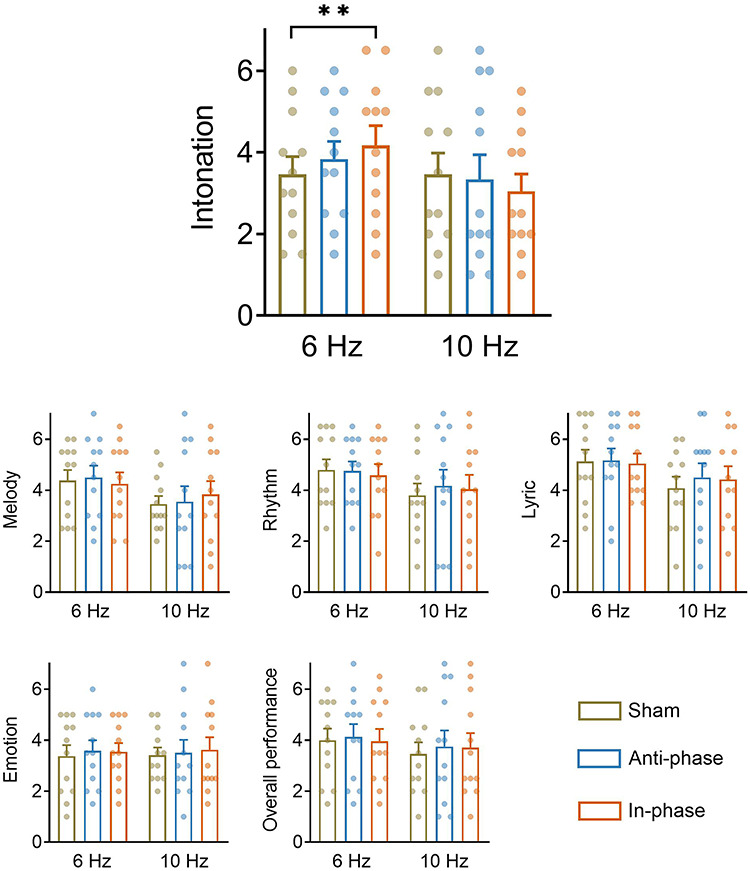
Learning performance ratings. Experts were asked to evaluate six aspects of learners’ solo performance (intonation, melody, rhythm, lyric, emotion and overall performance—see section Methods for details). Results revealed that 6 Hz in-phase dual brain stimulation led to better intonation compared to the sham stimulation. **P < 0.01. Error bar represents standard error. The other aspects of the performance were not affected by dual brain stimulation.

Experts’ ratings of the solo performances (provided following the learning session) are displayed on Figure 3. As hypothesized (see section Introduction), the ratings indicated that intonation accuracy changed as a function of the dual brain stimulation condition. Specifically, the results yielded evidence for an interaction between RELATIVE-PHASE and FREQUENCY, *F*_(2,44)_ = 6.86, *P* = 0.003: raters judged intonation to be better for songs associated with dual brain in-phase stimulation (4.17 ± 1.68) compared to sham stimulation (3.46 ± 1.50), *t*_(44)_ = 3.29, *P* = 0.006. Crucially, this was the case only for 6 Hz, but not 10 Hz, stimulation, *t*s < 1.94, *P*s > 0.14. There were no significant main effects, *F*s < 0.69, *P*s > 0.41. The mixed effects analyses conducted on the ratings associated with the other musical aspects yielded no statistically significant effects, *F*s < 2.25, *P*s > 0.11 (Figure 3).

### Interpersonal movement synchrony partially mediated the effect of dual brain stimulation on intonation learning performance

**Fig. 4:**
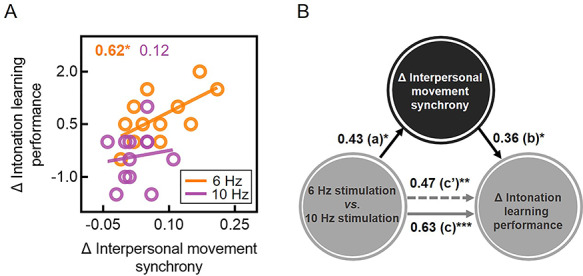
Correlation and mediation analyses. (A) Δ Interpersonal movement synchrony (in-phase minus sham) was positively correlated with Δ intonation learning performance in the 6 Hz (but not 10 Hz) group. (B) The effect of dual brain stimulation on Δ intonation learning performance (in-phase minus sham) is partially mediated by Δ interpersonal movement synchrony (in-phase minus sham). All path coefficients are standardized. *P < 0.05, **P < 0.01, ***P < 0.001.

The results from the correlation analysis are shown on Figure 4A. The results indicated that the stimulation-mediated enhancements (referred to as ‘Δ’) of interpersonal movement synchrony and intonation learning performance were positively correlated (*r* = 0.62, *P* = 0.03). This implied that 6 Hz in-phase dual brain stimulation led to relatively more synchronized movement in those learners whose performance was also rated higher. This observation suggested that dual brain stimulation had possibly enhanced the acquisition of the musical material by inducing spontaneous and synchronized movement across the instructor and the learner.

This hypothesis was confirmed by the results of the mediation analysis (Figure 4B). Notably, the results indicated (*i*) that dual brain stimulation could predict learning performance (path *c* = 0.63, *P* = 0.001) and (*ii*) that the relationship between dual brain stimulation and learning performance was reduced—although still significant—when interpersonal movement synchrony was included in the model as a mediator (path *c’* = 0.47, *P* = 0.004). This mediation effect was different from zero with 95% confidence (*β* = 0.26, confidence intervals = 0.01–0.74). Thus, interpersonal movement synchrony acted as a partial mediator of the effect of dual brain stimulation on intonation learning performance, possibly facilitating the effect of dual brain stimulation on learning.

## Discussion

We report unprecedented evidence that dual brain stimulation can augment social interactive learning. Alternating currents were delivered simultaneously through the brains of learners and instructors—engaged in a social learning task entailing the acquisition of a song—targeting inferior frontal cortical (IFC) regions. When the exogenously controlled currents were programmed to both oscillate at 6 Hz, and with an in-phase relation across the learner and the instructor, we observed enhanced learners’ performance. Specifically, intonation learning performance following 6 Hz in-phase stimulation was rated as higher than following conditions implying sham stimulation. This effect was phase and frequency dependent, as we did not observe comparable results in the 6 Hz anti-phase and 10 Hz control conditions.

This result is particularly important because it fits nicely with our previous correlational evidence showing that—in the context of a similar song-learning task—the brains of learners and instructors synchronize, and the strength of such synchronization predicts learners’ intonation learning performance (Pan *et al.,* 2018). Thus, going well beyond the previous observation, our current result indicates that inter-brain synchronization is not simply an epiphenomenon of social interactive learning, but can causally enhance social interactive learning. This result speaks to a large community of scientists working in hyperscanning research addressing, besides learning, other topics such as cooperation (Pan *et al.,* 2017), decision making (Hu *et al.,* 2018), communication (Jiang *et al.,* 2012) and joint attention (Goelman *et al.,* 2019). It shows that the notions acquired using hyperscanning can be brought to a whole new frontier. Rather than seeking correlational evidence between inter-brain synchrony and social behavior, scientists could attempt to manipulate social behavior by controlling inter-brain synchrony. This could lead to a noteworthy paradigm shift in social neuroscience, with potential applications touching on pedagogy, psychiatry or economics (Tang *et al.,* 2015; Leong and Schilbach, 2019; Redcay and Schilbach, 2019).

Dual brain stimulation has been firstly developed by Novembre *et al.* (2017) and later used by others (Szymanski *et al.,* 2017). In their first study, Novembre *et al.* (2017) targeted primary motor regions (M1) while two participants were preparing to tap their fingers in synchrony. It was reported that in-phase 20 Hz stimulation enhanced interpersonal coordination, specifically the dyad’s capacity to establish synchronized behavior. Based on our previous observations (Pan *et al.,* 2018) and other music-related neuroscientific reports (Osaka *et al.,* 2015), our study was not meant to target M1, but rather IFC. For this reason, the stimulation was delivered more frontally (approximately over FC5 instead of C3—see section Methods). Furthermore, the delivered currents oscillated in the theta range (6 Hz), as opposed to the beta range (20 Hz), in accordance with relevant literature characterizing the neuroanatomical origin of such neural rhythms (Cohen and Cavanagh, 2011; Feurra *et al.,* 2011; Cavanagh and Frank, 2014).

Nevertheless, because of the similar approach used in the two studies, we explored whether the two participants’ bodies swayed during the learning task, and whether those putative movements synchronized across learners and instructors. This was also inspired by previous work indicating that inter-brain synchronization in the theta band is associated with interpersonal imitation of movements (Dumas *et al.,* 2010). The reader should bear in mind that participants were not instructed to perform full-body movements. Thus, this analysis addressed spontaneous, as opposed to intentional (i.e. goal directed, Wen *et al.,* 2016), body movement, which is meant to be a functionally distinct cognitive and neurobiological process (Nusseck and Wanderley, 2009; Keller, 2014).

The analysis of interpersonal (spontaneous) movement synchrony yielded a very interesting result. Specifically, it showed how 6 Hz in-phase stimulation not only enhanced learning performance, but also led to enhanced movement synchrony between the learner and the instructor. Specifically, keeping in mind the structure of the experimental procedure, participants increased interpersonal movement synchrony while receiving 6 Hz in-phase dual brain stimulation and simultaneously learning the song (learning session). Next, following this specific stimulation condition, intonation learning performance was found to be enhanced (solo session).

This observation, and the temporal order of the effects, suggested that perhaps dual brain stimulation was not directly enhancing learning, but possibly it was doing so indirectly, i.e. through enhancement of interpersonal movement synchrony. This hypothesis was based on evidence indicating that interpersonal movement synchrony leads to noticeable pro-social effects such as enhanced partner likability, trust and affiliation (Hove and Risen, 2009; Wiltermuth and Heath, 2009; Keller *et al.,* 2014; Endedijk *et al.,* 2015; Reddish *et al.,* 2016; Hu *et al.,* 2017)—all factors that might impact upon an interpersonal learning performance (Bevilacqua *et al.,* 2019). To address this suggestion, we performed a mediation analysis. The results of this analysis provided some support to this account. Specifically, we observed that interpersonal movement synchrony worked as a partial mediator, and therefore, it could explain part of the effect of brain stimulation on learning.

It should be noted how the two proposed (not necessarily alternative) accounts of our results point towards markedly different underlying neurophysiological processes. The first account, according to which dual brain stimulation directly enhanced social learning, would be consistent with the broadly accepted notion that the phase of neural rhythms reflects periodic moments of enhanced cortical excitability (Sauseng and Klimesch, 2008; Schroeder and Lakatos, 2009). When such phase, recorded from the brain of one individual, aligns with the phase of another individual, the pair benefits from a neural alignment that might improve information transfer and support lots of interpersonal activities (Hasson and Frith, 2016). From this perspective, the instructor would have transferred information to the leaner more efficiently, as also suggested by some computational models (e.g. Dumas *et al.,* 2012; see also Hasson *et al.,* 2012 for a review). Instead, the account based on pro-social behavior being driven by enhanced movement synchrony could call for other neural processes, such as social affective networks (Fairhurst *et al.,* 2013), motivational- or reward-related neurocircuitry (Schilbach *et al.,* 2010; Pfeiffer *et al.,* 2014) or neurohormonal mechanisms regulating, e.g. endorphins or oxytocin release (Tarr *et al.,* 2014; Gebauer *et al.,* 2016). Future research might attempt to shed light upon the role (and possible interplay) of these mechanisms in social interactive learning.

A few other outstanding questions remain unanswered. For instance, besides affecting intonation accuracy specifically, our previous study reported also an effect of IBS on overall learning (Pan *et al.,* 2018). Why did our stimulation protocol affected intonation specifically, leaving overall performance unaffected? This difference could be explained by the different methodologies, and resulting timescales, used across our previous and current studies. Specifically, our IBS observations were made using fNIRS signals, which rely on hemodynamics and therefore unfold very slowly (Pan *et al.,* 2019), resulting in ultra-low frequencies (below 1 Hz). Instead, the current study relying on tACS was designed taking into account electrophysiological neural rhythms, which are much faster and normally range in between 1 and 100 Hz. In this area, 6 Hz is a promising rhythm due to its role in pitch processing (Behroozmand *et al.,* 2015), auditory change detection (Hsiao *et al.,* 2009) and interpersonal imitation of movements (Dumas *et al.,* 2010). It follows that the current approach might have specifically targeted neural mechanisms responsible for intonation, while the previous measure of IBS might have captured additional ones. Alternatively or complementarily to this explanation, it should be noted that intonation forms a central part of human vocal interaction and it is one of the most prominent features impacting upon the evaluation of musical performance (Morrison, 2000; Parncutt and McPherson, 2002).

A second point relates to sex composition of our cohort. Only female participants were tested in order to reduce variability of our sample, in accordance with previous evidence and recommendations (Cheng *et al.,* 2015; Baker *et al.,* 2016; Tang *et al.,* 2019). Although a strict criticism could question whether our results are generalizable to male individuals, we have no a priori reasons to expect so. Yet, being our effect sex-specific or sex-selective, we believe our results make a very important contribution to the emerging field of ‘second-person neuroscience’ (Schilbach *et al.,* 2013; Redcay and Schilbach, 2019).

Finally, alike most brain-stimulation studies, our data provide only a characterization of the behavioral consequences of brain stimulation, without a complementary description of the neurophysiological processes ongoing during stimulation. Our approach is certainly sufficient to (i) provide a causal relationship between a neural signal of interest and a resulting behavior or (ii) inspire interventions aimed to augment or potentially restore learning (Bolis *et al.,* 2017; Zhang *et al.,* 2018; Redcay and Schilbach, 2019; Pan and Cheng, 2020). However, when it comes to the interpretability of our research in the context of ongoing neurophysiological debates, it might be ideal to also obtain a brain measurement concurrently. Delivering tACS while measuring artifact-free EEG data is certainly challenging, but not impossible (Helfrich *et al.,* 2014). Thus, for the sake of completeness, and to increase the neurophysiological interpretability of the results, future studies might attempt to combine dual-tACS stimulation with e.g. dual-EEG.

## Conflict of interest

None declared.

## Funding

This work was supported by the National Natural Science Foundation of China (31872783) awarded to Y.H. and G.N., the National Natural Science Foundation of China (71942001), and the Basic Research Project of Shanghai Science and Technology Commission (19JC1410101).

## Supplementary Material

nsaa080_Supp

## References

[ref1a] Assaneo, M. F., Ripollés, P., Orpella, J., Lin, W. M., et al. (2019). Spontaneous synchronization to speech reveals neural mechanisms facilitating language learning. *Nature Neuroscience*, 22, 627–32. 10.1038/s41593-019-0353-z PMC6435400 30833700

[ref1] Babiloni F., Astolfi L. (2014). Social neuroscience and hyperscanning techniques: past, present and future. Neuroscience and Biobehavioral Reviews, 44, 76–93. 22917915 10.1016/j.neubiorev.2012.07.006 PMC3522775

[ref2] Baker J.M., Liu N., Cui X., et al. (2016). Sex differences in neural and behavioral signatures of cooperation revealed by fNIRS hyperscanning. Scientific Reports, 6, 26492. 27270754 10.1038/srep26492 PMC4897646

[ref3] Bates D., Maechler M., Bolker B., et al. (2015). Package ‘lme 4. Convergence, 12, 2.

[ref4] Behroozmand R., Ibrahim N., Korzyukov O., et al. (2015). Functional role of delta and theta band oscillations for auditory feedback processing during vocal pitch motor control. Frontiers in Neuroscience, 9, 109. 25873858 10.3389/fnins.2015.00109 PMC4379876

[ref5] Bevilacqua D., Davidesco I., Wan L., et al. (2019). Brain-to-brain synchrony and learning outcomes vary by student-teacher dynamics: evidence from a real-world classroom electroencephalography study. Journal of Cognitive Neuroscience, 31, 401–11. 29708820 10.1162/jocn_a_01274

[ref6] Bolis D., Balsters J., Wenderoth N., et al. (2017). Beyond autism: introducing the dialectical misattunement hypothesis and a Bayesian account of intersubjectivity. Psychopathology, 50, 355–72. 29232684 10.1159/000484353

[ref7] Cavanagh J.F., Frank M.J. (2014). Frontal theta as a mechanism for cognitive control. Trends in Cognitive Sciences, 18, 414–21. 24835663 10.1016/j.tics.2014.04.012 PMC4112145

[ref8] Chen Y., Matheson L.E., Sakata J.T. (2016). Mechanisms underlying the social enhancement of vocal learning in songbirds. Proceedings of National Academic Science USA., 113, 6641–6. 10.1073/pnas.1522306113 PMC4914165 27247385

[ref9] Cheng X., Li X., Hu Y. (2015). Synchronous brain activity during cooperative exchange depends on gender of partner: a fNIRS-based hyperscanning study. Human Brain Mapping, 36, 2039–48. 25691124 10.1002/hbm.22754 PMC6869051

[ref10] Cohen M.X., Cavanagh J.F. (2011). Single-trial regression elucidates the role of prefrontal theta oscillations in response conflict. Frontiers in Psychology, 2, 30. 21713190 10.3389/fpsyg.2011.00030 PMC3111011

[ref11] Crivelli-Decker J., Hsieh L.T., Clarke A., et al. (2018). Theta oscillations promote temporal sequence learning. Neurobiology of Learning and Memory, 153, 92–103. 29753784 10.1016/j.nlm.2018.05.001

[ref12] Dumas G., Nadel J., Soussignan R., et al. (2010). Inter-brain synchronization during social interaction. PloS ONE, 5, e12166. 20808907 10.1371/journal.pone.0012166 PMC2923151

[ref13] Dumas G., Lachat F., Martinerie J., et al. (2011). From social behaviour to brain synchronization: review and perspectives in hyperscanning. IRB, 32, 48–53.

[ref14] Dumas G., Chavez M., Nadel J., et al. (2012). Anatomical connectivity influences both intra-and inter-brain synchronizations. PloS ONE, 7, e36414. 22590539 10.1371/journal.pone.0036414 PMC3349668

[ref15] Endedijk H.M., Ramenzoni V.C., Cox R.F., et al. (2015). Development of interpersonal coordination between peers during a drumming task. Developmental Psychology, 51, 714–21. 25775110 10.1037/a0038980

[ref16] Fairhurst M.T., Janata P., Keller P.E. (2013). Being and feeling in sync with an adaptive virtual partner: brain mechanisms underlying dynamic cooperativity. Cerebral Cortex, 23, 2592–600. 22892422 10.1093/cercor/bhs243

[ref17] Feurra M., Bianco G., Santarnecchi E., et al. (2011). Frequency-dependent tuning of the human motor system induced by transcranial oscillatory potentials. The Journal of Neuroscience, 31, 12165–70. 21865459 10.1523/JNEUROSCI.0978-11.2011 PMC6623220

[ref18] Gebauer L., Witek M.A.G., Hansen N.C., et al. (2016). Oxytocin improves synchronisation in leader-follower interaction. Scientific Reports, 6, 38416. 27929100 10.1038/srep38416 PMC5144006

[ref19] Goelman G., Dan R., Stößel G., et al. (2019). Bidirectional signal exchanges and their mechanisms during joint attention interaction–a hyperscanning fMRI study. Neuro Image., 198, 242–54. 31112784 10.1016/j.neuroimage.2019.05.028

[ref20] Hasson U., Frith C.D. (2016). Mirroring and beyond: coupled dynamics as a generalized framework for modelling social interactions. Philosophical Transactions of the Royal Society B, 371, 20150366. 10.1098/rstb.2015.0366 PMC4843605 27069044

[ref21] Hasson U., Ghazanfar A.A., Galantucci B., et al. (2012). Brain-to-brain coupling: a mechanism for creating and sharing a social world. Trends in Cognitive Sciences, 16, 114–21. 22221820 10.1016/j.tics.2011.12.007 PMC3269540

[ref22] Helfrich R.F., Schneider T.R., Rach S., et al. (2014). Entrainment of brain oscillations by transcranial alternating current stimulation. Current Biology, 24, 333–9. 24461998 10.1016/j.cub.2013.12.041

[ref23] Holland R., Leff A.P., Josephs O., et al. (2011). Speech facilitation by left inferior frontal cortex stimulation. Current Biology, 21, 1403–7. 21820308 10.1016/j.cub.2011.07.021 PMC3315006

[ref24] Holland R., Leff A.P., Penny W.D., et al. (2016). Modulation of frontal effective connectivity during speech. Neuro Image., 140, 126–33. 26825443 10.1016/j.neuroimage.2016.01.037 PMC5033642

[ref25] Hove M.J., Risen J.L. (2009). It’s all in the timing: interpersonal synchrony increases affiliation. Social Cognition, 27, 949–60.

[ref26] Hsiao F.K., Wu Z.A., Ho L.T., et al. (2009). Theta oscillation during auditory change detection: an MEG study. Biological Psychology, 81, 58–66. 19428969 10.1016/j.biopsycho.2009.01.007

[ref27] Hu Y., Hu Y., Li X., et al. (2017). Brain-to-brain synchronization across two persons predicts mutual prosociality. Social Cognitive and Affective Neuroscience, 12, 1835–44. 29040766 10.1093/scan/nsx118 PMC5716073

[ref28] Hu Y., Pan Y., Shi X., et al. (2018). Inter-brain synchrony and cooperation context in interactive decision making. Biological Psychology, 133, 54–62. 29292232 10.1016/j.biopsycho.2017.12.005

[ref29] Jiang J., Dai B., Peng D., et al. (2012). Neural synchronization during face-to-face communication. The Journal of Neuroscience, 32, 16064–9. 23136442 10.1523/JNEUROSCI.2926-12.2012 PMC6621612

[ref30] Keller, PE. (2014). Ensemble performance: Interpersonal alignment of musical expression. Expressiveness in music performance: empirical approaches across styles and cultures (eds, Fabian D, Timmers R & Schubert E), pp. 260–82. Oxford, UK: Oxford University Press.

[ref31] Keller P.E., Novembre G., Hove M.J. (2014). Rhythm in joint action: psychological and neurophysiological mechanisms for real-time interpersonal coordination. Philosophical Transactions of the Royal Society B, 369, 20130394. 10.1098/rstb.2013.0394 PMC4240961 25385772

[ref32] Kleinbub J. R., Ramseyer F. (2018). rMEA: Synchrony in motion energy analysis (MEA) time-series. Available: https://CRAN.R-project.org/package=rMEA (Accessed on April 10, 2020).

[ref33] Klimesch W. (2012). Alpha-band oscillations, attention, and controlled access to stored information. Trends in Cognitive Sciences, 16, 606–17. 23141428 10.1016/j.tics.2012.10.007 PMC3507158

[ref34] Kupper Z., Ramseyer F., Hoffmann H., et al. (2015). Nonverbal synchrony in social interactions of patients with schizophrenia indicates socio-communicative deficits. PLoS ONE, 10, e0145882. 26716444 10.1371/journal.pone.0145882 PMC4696745

[ref35] Kuznetsova A., Brockhoff P.B., Christensen R.H.B. (2017). Lmer test package: tests in linear mixed effects models. Journal of Statistical Software, 82, 1–26. 10.18637/jss.v082.i13.

[ref36] Lee C., Jung Y.J., Lee S.J., et al. (2017). COMETS2: an advanced MATLAB toolbox for the numerical analysis of electric fields generated by transcranial direct current stimulation. Journal of Neuroscience Methods, 277, 56–62. 27989592 10.1016/j.jneumeth.2016.12.008

[ref37] Lenth R. V. (2018). emmeans: Estimated Marginal Means, aka Least Squares Means. R package version 1.1. Available: https://CRAN.R-project.org/package=emmeans (Accessed on April 10, 2020).

[ref38] Leong V., Schilbach L. (2019). The promise of two-person neuroscience for developmental psychiatry: using interaction-based sociometrics to identify disorders of social interaction. The British Journal of Psychiatry, 215, 636–8. 31014406 10.1192/bjp.2019.73

[ref39] Lindenberger U., Li S.C., Gruber W., et al. (2009). Brains swinging in concert: cortical phase synchronization while playing guitar. BMC Neuroscience, 10, 22. 19292892 10.1186/1471-2202-10-22 PMC2662862

[ref40] Liu Y., Piazza E.A., Simony E., et al. (2017). Measuring speaker–listener neural coupling with functional near infrared spectroscopy. Scientific Reports, 7, 43293. 28240295 10.1038/srep43293 PMC5327440

[ref41] Liu J., Zhang R., Geng B., et al. (2019). Interplay between prior knowledge and communication mode on teaching effectiveness: interpersonal neural synchronization as a neural marker. Neuro Image., 193, 93–102. 30851445 10.1016/j.neuroimage.2019.03.004

[ref42] Luke S.G. (2017). Evaluating significance in linear mixed-effects models in R. Behavior Research and Methods, 49, 1494–502. 10.3758/s13428-016-0809-y 27620283

[ref43] Mac Kinnon D.P., Fairchild A.J., Fritz M.S. (2007). Mediation analysis. Annual Reviews of Psychology, 58, 593–614. 10.1146/annurev.psych.58.110405.085542 PMC2819368 16968208

[ref44] Mandell J., Schulze K., Schlaug G. (2007). Congenital amusia: An auditory-motor feedback disorder. Restorative Neurology and Neuroscience, 25, 323–34. 17943009

[ref45] Maris E., Oostenveld R. (2007). Nonparametric statistical testing of EEG-and MEG-data. Journal of Neuroscience Methods, 164, 177–90. 17517438 10.1016/j.jneumeth.2007.03.024

[ref46] Montague P.R., Berns G.S., Cohen J.D., et al. (2002). Hyperscanning: simultaneous fMRI during linked social interactions. Neuro Image., 16, 1159–64. 12202103 10.1006/nimg.2002.1150

[ref47] Morrison S.J. (2000). Effect of melodic context, tuning behaviors, and experience on the intonation accuracy of wind players. Journal of Research in Music Education, 48, 39–51.

[ref48] Nastase S.A., Gazzola V., Hasson U., et al. (2019). Measuring shared responses across subjects using intersubject correlation. Social Cognitive and Affective Neuroscience, 14, 667–85. 31099394 10.1093/scan/nsz037 PMC6688448

[ref49] Novembre G., Knoblich G., Dunne L., et al. (2017). Interpersonal synchrony enhanced through 20 Hz phase-coupled dual brain stimulation. Social Cognitive and Affective Neuroscience, 12, 662–70. 10.1093/scan/nsw172 PMC5390732 28119510

[ref50] Novembre G., Mitsopoulos Z., Keller P.E. (2019). Empathic perspective taking promotes interpersonal coordination through music. Scientific Reports, 9, 12255. 31439866 10.1038/s41598-019-48556-9 PMC6706439

[ref51] Nusseck M., Wanderley M.M. (2009). Music and motion—how music-related ancillary body movements contribute to the experience of music. Music Perception, 26, 335–53.

[ref52] Osaka N., Minamoto T., Yaoi K., et al. (2015). How two brains make one synchronized mind in the inferior frontal cortex: fNIRS-based hyperscanning during cooperative singing. Frontiers in Psychology, 6, 1811. 26635703 10.3389/fpsyg.2015.01811 PMC4659897

[ref53] Pan Y., Cheng X. (2020). Two-person approaches to studying social interaction in psychiatry: uses and clinical relevance. Frontiers in Psychiatry, 11, 301. 32390881 10.3389/fpsyt.2020.00301 PMC7193689

[ref54] Pan Y., Cheng X., Zhang Z., et al. (2017). Cooperation in lovers: an fNIRS-based hyperscanning study. Human Brain Mapping, 38, 831–41. 27699945 10.1002/hbm.23421 PMC6867051

[ref55] Pan Y., Novembre G., Song B., et al. (2018). Interpersonal synchronization of inferior frontal cortices tracks social interactive learning of a song. Neuro Image., 183, 280–90. 30086411 10.1016/j.neuroimage.2018.08.005

[ref56] Pan Y., Borragán G., Peigneux P. (2019). Applications of functional near-infrared spectroscopy in fatigue, sleep deprivation, and social cognition. Brain Topography, 32, 998–1012. 31664637 10.1007/s10548-019-00740-w

[ref57] Pan Y., Dikker S., Goldstein P., et al. (2020). Instructor-learner brain coupling discriminates between instructional approaches and predicts learning. Neuro Image., 211, 116657. 32068165 10.1016/j.neuroimage.2020.116657

[ref58] Parncutt R., McPherson G. (2002). The Science and Psychology of Music Performance: Creative Strategies for Teaching and Learning, Oxford: Oxford University Press.

[ref59] Pfeiffer U.J., Schilbach L., Timmermans B., et al. (2014). Why we interact: on the functional role of the striatum in the subjective experience of social interaction. Neuro Image., 101, 124–37. 24996121 10.1016/j.neuroimage.2014.06.061

[ref60] Preacher K.J., Hayes A.F. (2008). Asymptotic and resampling strategies for assessing and comparing indirect effects in multiple mediator models. Behavior Research and Methods, 40, 879–91. 10.3758/brm.40.3.879 18697684

[ref61] R Core Team (2020). R: A Language and Environment for Statistical Computing, Vienna, Austria: R Foundation for Statistical Computing URL: https://www.R-project.org/.

[ref63] Ramseyer F., Tschacher W. (2011). Nonverbal synchrony in psychotherapy: coordinated body movement reflects relationship quality and outcome. Journal of Consulting and Clinical Psychology, 79, 284–95. 21639608 10.1037/a0023419

[ref64] Redcay E., Schilbach L. (2019). Using second-person neuroscience to elucidate the mechanisms of social interaction. Nature Reviews. Neuroscience, 20, 495–505. 31138910 10.1038/s41583-019-0179-4 PMC6997943

[ref65] Reddish P., Tong E.M., Jong J., et al. (2016). Collective synchrony increases prosociality towards non-performers and outgroup members. The British Journal of Social Psychology, 55, 722–38. 27683102 10.1111/bjso.12165

[ref66] Sänger J., Müller V., Lindenberger U. (2012). Intra-and interbrain synchronization and network properties when playing guitar in duets. Frontiers in Human Neuroscience, 6, 312. 23226120 10.3389/fnhum.2012.00312 PMC3509332

[ref67] Sauseng P., Klimesch W. (2008). What does phase information of oscillatory brain activity tell us about cognitive processes? Neuroscience and Biobehavioral Reviews, 32, 1001–13. 18499256 10.1016/j.neubiorev.2008.03.014

[ref68] Schilbach L., Wilms M., Eickhoff S.B., et al. (2010). Minds made for sharing: initiating joint attention recruits reward-related neurocircuitry. Journal of Cognitive Neuroscience, 22, 2702–15. 19929761 10.1162/jocn.2009.21401

[ref69] Schilbach L., Timmermans B., Reddy V., et al. (2013). Toward a second-person neuroscience. The Behavioral and Brain Sciences, 36, 393–414. 23883742 10.1017/S0140525X12000660

[ref70] Schroeder C.E., Lakatos P. (2009). Low-frequency neuronal oscillations as instruments of sensory selection. Trends in Neurosciences, 32, 9–18. 19012975 10.1016/j.tins.2008.09.012 PMC2990947

[ref71] Szymanski C., Müller V., Brick T.R., et al. (2017). Hyper-transcranial alternating current stimulation: experimental manipulation of inter-brain synchrony. Frontiers in Human Neuroscience, 11, 539. 29167638 10.3389/fnhum.2017.00539 PMC5682643

[ref72] Takeuchi N., Mori T., Suzukamo Y., et al. (2017). Integration of teaching processes and learning assessment in the prefrontal cortex during a video game teaching–learning task. Frontiers in Psychology, 7, 2052. 28119650 10.3389/fpsyg.2016.02052 PMC5220187

[ref73] Tang H., Mai X., Wang S., et al. (2015). Interpersonal brain synchronization in the right temporo-parietal junction during face-to-face economic exchange. Social Cognitive and Affective Neuroscience, 11, 23–32. 26211014 10.1093/scan/nsv092 PMC4692317

[ref74] Tang H., Zhang S., Jin T., et al. (2019). Brain activation and adaptation of deception processing during dyadic face-to-face interaction. Cortex, 120, 326–39. 31401400 10.1016/j.cortex.2019.07.004

[ref75] Tarr B., Launay J., Dunbar R.I. (2014). Music and social bonding: “self-other” merging and neurohormonal mechanisms. Frontiers in Psychology, 5, 1096. 25324805 10.3389/fpsyg.2014.01096 PMC4179700

[ref76] Tschacher W., Rees G.M., Ramseyer F. (2014). Nonverbal synchrony and affect in dyadic interactions. Frontiers in Psychology, 5, 1323. 25505435 10.3389/fpsyg.2014.01323 PMC4241744

[ref77] Verga L., Kotz S.A. (2019). Spatial attention underpins social word learning in the right fronto-parietal network. Neuro Image., 195, 165–73. 30946951 10.1016/j.neuroimage.2019.03.071

[ref78] Wen W., Muramatsu K., Hamasaki S., et al. (2016). Goal-directed movement enhances body representation updating. Frontiers in Human Neuroscience, 10, 329. 27445766 10.3389/fnhum.2016.00329 PMC4923246

[ref79] Wiltermuth S.S., Heath C. (2009). Synchrony and cooperation. Psychological Science, 20, 1–5. 19152536 10.1111/j.1467-9280.2008.02253.x

[ref80] Wischnewski M., Zerr P., Schutter D.J. (2016). Effects of theta transcranial alternating current stimulation over the frontal cortex on reversal learning. Brain Stimulation, 9, 705–11. 27162098 10.1016/j.brs.2016.04.011

[ref81] Zhang Y., Meng T., Hou Y., et al. (2018). Interpersonal brain synchronization associated with working alliance during psychological counseling. Psychiatry Research: Neuroimaging, 282, 103–9. 30292535 10.1016/j.pscychresns.2018.09.007

[ref82] Zheng L., Chen C., Liu W., et al. (2018). Enhancement of teaching outcome through neural prediction of the students’ knowledge state. Human Brain Mapping, 39, 3046–57. 29575392 10.1002/hbm.24059 PMC6866636

