# Supplementary material for: Dual brain stimulation enhances interpersonal learning through spontaneous movement synchrony
Source: Soc Cogn Affect Neurosci. 2020 Jun 15;16(1-2):210–21. doi: 10.1093/scan/nsaa080 (PMC7812617; doi:10.1093/scan/nsaa080)
Supplement: nsaa080_Supp [file nsaa080_supp.zip › scan-20-087-File006_nsaa080.docx]

*Supplementary Material*

**Dual brain stimulation enhances interpersonal learning through spontaneous movement synchrony**

Yafeng Pan^1,2,6+^, Giacomo Novembre^3,4+^, Bei Song^1,5^, Yi Zhu^1^, and Yi Hu^1*^

*^1^ School of Psychology and Cognitive Science, East China Normal University, 200062 Shanghai, People’s Republic of China*

*^2^ Neuropsychology and Functional Neuroimaging Research Unit (UR2NF), Université Libre de Bruxelles, B-1050 Bruxelles, Belgium*

*^3^ Department of Neuroscience, Physiology and Pharmacology, University College London, WC1E 6BT London, United Kingdom*

*^4^ Neuroscience and Behaviour Laboratory, Istituto Italiano di Tecnologia, 00161 Rome, Italy*

*^5^ Department of Musicology, Harbin Conservatory of Music, 150070 Heilongjiang, People’s Republic of China*

*^6^ Department of Clinical Neuroscience, Karolinska Institutet, 17165 Stockholm, Sweden*

**^+^** These authors made equal contributions.

**^*^ Corresponding author:**

Yi Hu: [yhu@psy.ecnu.edu.cn](mailto:yhu@psy.ecnu.edu.cn)

**Supplementary Text**

**Brain stimulation questionnaires.** In a subset of participants (n = 12), we tested whether participants could detect the brain stimulation. Results were not consistent with this hypothesis. When asked whether they could or could not perceive the stimulation following each block, participants were at chance level (52.8%), indicating that participants could not reliably distinguish sham vs. actual stimulation. Side-effects were comparable across conditions (*F*s < 1.14, *P*s > 0.12). A subset of participants (n = 3) reported perceiving phosphenes (none of them were paired together).

**Pesudosynchrony control analysis.** To control for spurious synchrony, we additionally conducted a pseudosynchrony analysis consisting of three main steps (cf. Ramseyer and Tschacher, 2011). First, we generated surrogate datasets by using an automated shuffling algorithm (Kleinbub and Ramseyer, 2018). This consisted of randomly pairing learners and instructors (*N* = 100 out of each genuine dataset) (Kleinbub and Ramseyer, 2018; Ramseyer and Tschacher, 2011). Second, pseudosynchrony was estimated using cross-correlation (as for the original data). Finally, the value representing genuine movement synchrony (cross-correlation coefficients, averaged across all lags; Ramseyer and Tschacher, 2011) was compared with the value representing pseudosynchrony (note that each dyad was characterized by one single value of genuine movement synchrony and, correspondingly, one value of pseudosynchrony). We used multi-level (mixed effects) modeling to control for the non-independence of some of the data points (see $$ section from the manuscript). Synchrony values were modeled by DATASET TYPE (genuine vs. pseudo). Furthermore, LEARNER and INSTRUCTOR were specified as random effects (with the former being nested within the latter). The results indicated that genuine movement synchrony (*M* ± *SD*, 0.14 ± 0.02) was significantly higher than pseudosynchrony (*M* ± *SD*, 0.13 ± 0.01). *F*_(1,43)_ = 11.58, *P* = 0.001_。_ This confirmed that the movement synchrony measured across learners and instructors did not emerge by chance.

Having established the difference between genuine synchrony and pseudosynchrony, we next sought to compare synchrony across different conditions and groups. To do so, we first standardized the movement synchrony using the following formula:

$$R_{\mathrm{zscore}}= \frac{genuine synchrony- M_{\mathrm{pseudosynchrony}}}{\mathrm{SD}_{\mathrm{pseudosynchrony}}}$$

The z-transformed values were submitted to a multi-level (mixed effects) model: we treated RELATIVE PHASE and FREQUENCY as fixed effects. Instead, LEARNER and INSTRUCTOR were treated as random effects. Because each instructor was paired with several learners, the variable LEARNER was nested within the variable INSTRUCTOR. In line with our main findings, we observed main effects of RELATIVE PHASE, *F*_(2,44)_ = 5.36, *P* = 0.008, FREQUENCY, *F*_(1,19)_ = 3.42, *P* = 0.08, and an interaction, *F*_(2,44)_ = 5.60, *P* = 0.007. Following up on the interaction, we examined the effect of RELATIVE PHASE separately by FREQUENCY. In the 6 Hz group, the in-phase condition elicited larger standardized movement synchrony than the sham condition, *t*_(44)_ = 4.34, *P* = 0.0002. Synchrony for the anti-phase and sham conditions did not differ from one another, *t*_(44)_ = 0.87, *P* = 0.66. In the 10 Hz group, no significant effects were revealed, *t*s < 0.87, *P*s > 0.66.

**Instructor-specific effect.** We repeated the mediation analyses by adding INSTRUCTOR as a covariate. This compensatory analysis yielded results that were fully in line with previous findings: path *a* = 0.43, *P* = 0.02; path *b* = 0.37, *P* = 0.02; path *c’* = 0.47, *P* = 0.004; path *c* = 0.63, *P* = 0.001 (standardized coefficients). The path coefficient relating the covariate (INSTRUCTOR) to the dependent variable (Δ intonation learning performance) was not significant (standardized *β* = 0.09, *P* = 0.56).

**Phase stability test.** We conducted a control experiment to test the phase alignment of the electric currents generated by the two stimulators (across all conditions) for a duration equal to the duration of our trials (i.e. 8 minutes). The results from this experiment indicate that the outputted currents were well aligned and that the phase shift was negligible.

Specifically, we analyzed the data from the four stimulation conditions (6 Hz in-phase, 6 Hz anti-phase, 10 Hz in-phase, and 10 Hz anti-phase) by computing the relative phase and the phase-locking value (PLV) between the two outputted signals. Phases and amplitudes were extracted using the Hilbert transform.

During in-phase stimulation (both 6 and 10 Hz), the average relative phase was extremely close to 0° (specifically, it ranged between 0.90° and 1.66°), while during anti-phase stimulation (both 6 and 10 Hz), the average relative phase was extremely close to 180° (specifically, it ranged between 178.40° and 179.90°) (see **Figure S1** below).

To address the issue of a potential gradual phase shift, we computed a PLV index according to the function below.

${PLV}_{i,k}=T^{-1}\left| \sum_{t=1}^{N} {exp}^{j\left( \varphi_{i}\left( t \right)-\varphi_{k}\left( t \right) \right)} \right|$,

where *T* represents the time points, $\varphi$ is the phase and | | represents the complex modulus. *i* and *k* indicate the recordings from tACS #1 and tACS #2 in a dual brain stimulation system, respectively. The PLV ranges from 0 to 1, where 1 indicates that the relative phase between signals is constant across the whole time window.

Again, as it should be expected, the PLVs were extremely close to 1 in all conditions. Specifically, the PLV ranged from 0.9976 to 0.9998 (**Fig. S1**). These results confirmed that the relative phase across the two different stimulators was constant.


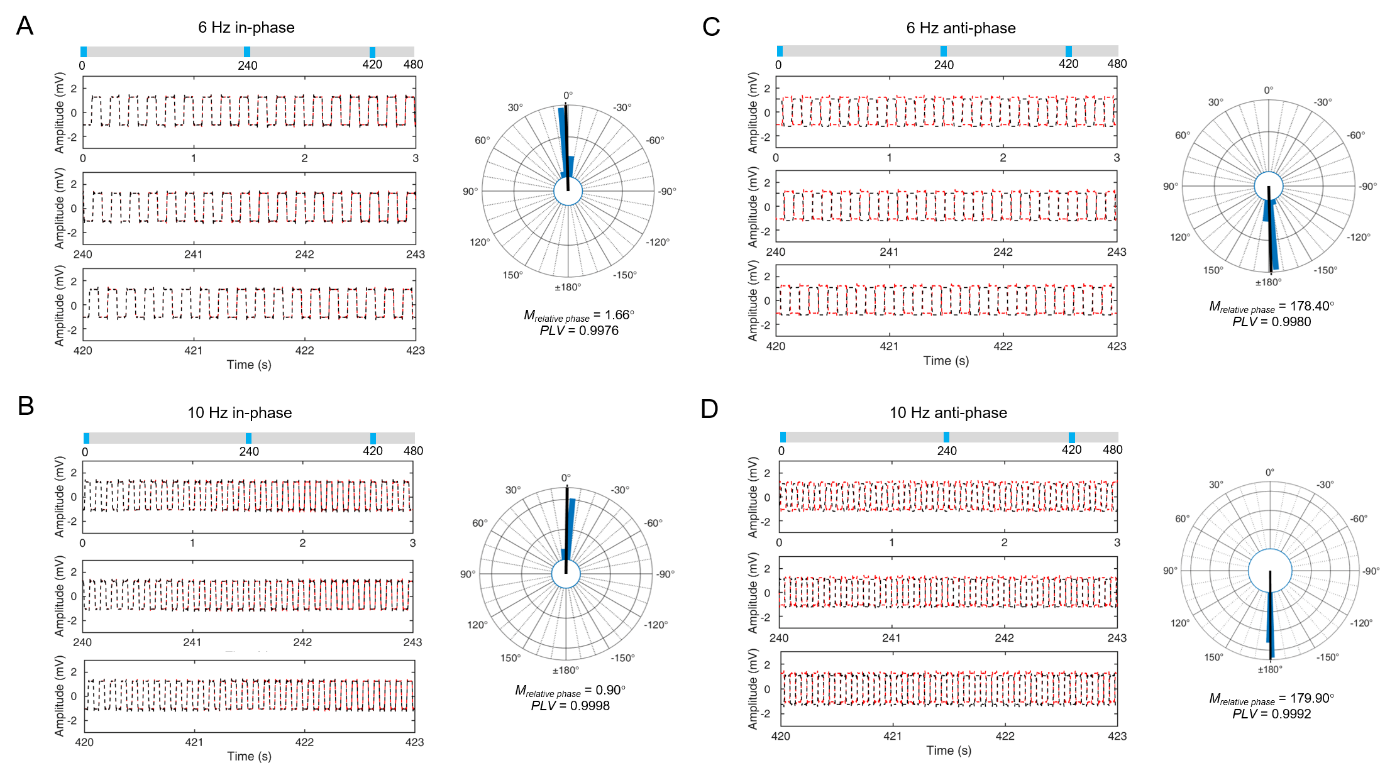


**Fig. S1.** Results from the control experiment. The time-series represent the alternating current generated by the two stimulators (red and black dashed lines), and their relative phase distribution, from three different time windows within a trial (i.e., 0-3 s, 240-243 s, and 420-423 s, representing early, middle, and late time segments within a trial, respectively). Top: 6 Hz stimulation; Bottom: 10 Hz stimulation; Left: in-phase stimulation; Right: anti-phase stimulation.

**Table S1.** Difference in pitch discrimination accuracy across the 6 Hz and 10 Hz groups.

| 6 Hz | | 10 Hz | | *t* | *P* | *Cohen’s d* |
| --- | --- | --- | --- | --- | --- | --- |
| *Mean* | *SD* | *Mean* | *SD* |  |  |  |
| 68.5% | 0.08 | 66.4% | 0.10 | 0.57 | 0.58 | 0.23 |

*Note*. Percentage scores were obtained from <http://jakemandell.com/tonedeaf/>.

**Table S2.** Counterbalancement of different stimulation frequencies (6 Hz and 10 Hz), relative-phases (in-phase, anti-phase, and sham), learning contents (songs), and instructor across experimental blocks and dyads.

| ***Dyad*** | ***Instructor*** | ***Block 1*** | ***Block 2*** | ***Block 3*** |
| --- | --- | --- | --- | --- |
| 1 | 1 | 6 Hz/in-phase/song 1 | 6 Hz/anti-phase/song 2 | 6 Hz/sham/song 3 |
| 2 | 1 | 6 Hz/ in-phase/song 2 | 6 Hz/sham/song 1 | 6 Hz/anti-phase/song 3 |
| 3 | 1 | 6 Hz/anti-phase/song 1 | 6 Hz/in-phase/song 3 | 6 Hz/sham/song 2 |
| 4 | 2 | 6 Hz/anti-phase/song 2 | 6 Hz/sham/song 3 | 6 Hz/in-phase/song 1 |
| 5 | 2 | 6 Hz/sham/song 1 | 6 Hz/anti-phase/song 3 | 6 Hz/in-phase/song 2 |
| 6 | 2 | 6 Hz/sham/song 2 | 6 Hz/in-phase/song 3 | 6 Hz/anti-phase/song 1 |
| 7 | 3 | 6 Hz/in-phase/song 1 | 6 Hz/anti-phase/ song 2 | 6 Hz/sham/song 3 |
| 8 | 3 | 6 Hz/ in-phase/song 2 | 6 Hz/sham/song 1 | 6 Hz/anti-phase/song 3 |
| 9 | 3 | 6 Hz/anti-phase/song 1 | 6 Hz/in-phase/song 3 | 6 Hz/sham/song 2 |
| 10 | 4 | 6 Hz/anti-phase/song 2 | 6 Hz/sham/song 3 | 6 Hz/in-phase/song 1 |
| 11 | 4 | 6 Hz/sham/song 1 | 6 Hz/anti-phase/song 3 | 6 Hz/in-phase/song 2 |
| 12 | 4 | 6 Hz/sham/song 2 | 6 Hz/in-phase/song 3 | 6 Hz/anti-phase/song 1 |
| 13 | 1 | 10 Hz/in-phase/song 1 | 10 Hz/anti-phase/ song 2 | 10 Hz/sham/song 3 |
| 14 | 1 | 10 Hz/ in-phase/song 2 | 10 Hz/sham/song 1 | 10 Hz/anti-phase/song 3 |
| 15 | 1 | 10 Hz/anti-phase/song 1 | 10 Hz/in-phase/song 3 | 10 Hz/sham/song 2 |
| 16 | 2 | 10 Hz/anti-phase/song 2 | 10 Hz/sham/song 3 | 10 Hz/in-phase/song 1 |
| 17 | 2 | 10 Hz/sham/song 1 | 10 Hz/anti-phase/song 3 | 10 Hz/in-phase/song 2 |
| 18 | 2 | 10 Hz/sham/song 2 | 10 Hz/in-phase/song 3 | 10 Hz/anti-phase/song 1 |
| 19 | 3 | 10 Hz/in-phase/song 1 | 10 Hz/anti-phase/ song 2 | 10 Hz/sham/song 3 |
| 20 | 3 | 10 Hz/ in-phase/song 2 | 10 Hz/sham/song 1 | 10 Hz/anti-phase/song 3 |
| 21 | 3 | 10 Hz/anti-phase/song 1 | 10 Hz/in-phase/song 3 | 10 Hz/sham/song 2 |
| 22 | 4 | 10 Hz/anti-phase/song 2 | 10 Hz/sham/song 3 | 10 Hz/in-phase/song 1 |
| 23 | 4 | 10 Hz/sham/song 1 | 10 Hz/anti-phase/song 3 | 10 Hz/in-phase/song 2 |
| 24 | 4 | 10 Hz/sham/song 2 | 10 Hz/in-phase/song 3 | 10 Hz/anti-phase/song 1 |

**Table S3.** Music performance evaluation criteria

|  |  | **Score** | | | | | | |
| --- | --- | --- | --- | --- | --- | --- | --- | --- |
| **Aspects** | **Criteria** | **Very high** | **High** | **Medium-high** | **Medium** | **Medium-low** | **Low** | **Very Low** |
| Intonation | Ability to accurately express sounds as “higher” and “lower” in the sense associated with musical melodies. | 7 | 6 | 5 | 4 | 3 | 2 | 1 |
| Melody | Ability to accurately express the linear succession of musical tones. | 7 | 6 | 5 | 4 | 3 | 2 | 1 |
| Rhythm | Effective expression of the timing of musical sounds and silences that occur over time. | 7 | 6 | 5 | 4 | 3 | 2 | 1 |
| Lyric | Accuracy of the singing lyrics. | 7 | 6 | 5 | 4 | 3 | 2 | 1 |
| Emotion | Ability to effectively express the emotion of the song. Signs of high ability include emotional facial and vocal expression. | 7 | 6 | 5 | 4 | 3 | 2 | 1 |
| Overall Performance | Overall ability to perform the music song. | 7 | 6 | 5 | 4 | 3 | 2 | 1 |
